# Supplementary material for: Content-rich biological network constructed by mining PubMed abstracts
Source: BMC Bioinformatics. 2004 Oct 8;5:147. doi: 10.1186/1471-2105-5-147 (PMC528731; doi:10.1186/1471-2105-5-147)
Supplement: Additional File 2 — The original results of the above study (non-essential files are deleted to keep the file size under the limit set by BMC bioinformatics). [file 1471-2105-5-147-S2.bz2 › chilibotAdditionalFile2/dip30/683ID10906130E17998/html/RPOA_RPOB.html]

 


 **RPOA** and **RPOB** 
  
Found 22 abstracts in PubMed, retrieved 22.  
 

 What does Google say? 
 PDF only 
| .edu only 

---

**Interactive relationship** (e.g. stimulation, inhibition, etc)

**Inhibitory relationship**- The lack of a PEP  [ **RPOA** ]  promoter in these species coincides with the loss of two genes that encode subunits of PEP  [ **RPOA** ]   **rpoA**  and  **rpoB** .  Ref: 12624769 Planta, 2003
**Neutral relationship**- We report here that atpB, an ATPase subunit gene has promoters for both NEP and PEP  [ **RPOA** ] , while clpP, a protease subunit gene, and the  **rpoB**  operon, encoding three PEP  [ **RPOA** ]  subunit genes, are exclusively transcribed from NEP promoters.  Ref: 9618584 Curr Genet, 1998
- Therefore,  **rpoA** ,  **rpoB** , rpoC1, and rpoC2 encode PEP  [ **RPOA** ]  subunits that are not essential components of the NEP transcription machinery.  Ref: 9701572 Plant Physiol, 1998
- The plastid  **rpoA** ,  **rpoB** , rpoC1, and rpoC2 genes encode subunits of the plastid encoded plastid RNA polymerase PEP  [ **RPOA** ] , an Escherichia coli like core enzyme.  Ref: 9701572 Plant Physiol, 1998

**Non-interactive relationship** (e.g. studied together, co-existance, homology, etc.)

- Amino terminal amino acid sequences of the 18 20, and 38 kDa polypeptides have been determined and found to correspond precisely to the sequences deduced from the 5 ends of the maize chloroplast rpoC2,  **rpoB** , and  **rpoA**  genes, respectively.  Ref: 2304916 Proc Natl Acad Sci U S A, 1990
- Furthermore, our data indicate that no functional copy of  **rpoA** ,  **rpoB** , rpoC1, or rpoC2 that could complement the deleted plastid rpo genes exists outside the plastids.  Ref: 9701572 Plant Physiol, 1998
- The genes,  **rpoA** ,  **rpoB**  and rpoC of Escherichia coli, which encode the RNA polymerase alpha, beta and beta subunits, respectively, have been individually placed on expression plasmids under the control of the bacteriophage T7 promoter.  Ref: 2197183 Gene, 1990
- Fusion proteins for chloroplast homologs of the RNA polymerase alpha  **rpoA** , beta  **rpoB** , and beta rpoC subunits were obtained from these genes.  Ref: 3049574 J Biol Chem, 1988
- Northern analysis of  **rpoA** ,  **rpoB** , rpoC, and six ribosomal protein genes indicated induction of transcription as well as antitermination as part of the mechanism of the regulation of gene expression.  Ref: 11681206 Proteomics, 2001
- Plastid genomes contain  **rpoA** ,  **rpoB** , rpoC1, and rpoC2 coding for alpha, beta, beta, and beta RNA polymerase subunits that are homologous to the alpha, beta, and beta subunits that constitute the core moiety of RNA polymerase in bacteria.  Ref: 8159791 Plant Physiol, 1994
- In order to examine the expression of the rpoBC genes in response to varied intracellular concentrations of the RNA polymerase subunits, a series of expression plasmids carrying the  **rpoB** , rpoC,  **rpoA**  alpha and rpoD sigma 70 genes in different combinations were then introduced into these cells.  Ref: 8830239 Mol Microbiol, 1996
- Sigma 38 has not effects on the product of  **rpoA** ,  **rpoB** , rpoC, groE and tu gene, depress the transcription of crp and promote the expression of rmf.  Ref: 9639823 Wei Sheng Wu Xue Bao, 1996
- Plastid run on assays and Northern analyses were employed to analyse gene expression in tobacco mutant plastids lacking the PEP  [ **RPOA** ]  genes  **rpoA** ,  **rpoB**  or rpoC1.  Ref: 10954088 Mol Gen Genet, 2000
- The chloroplast genome contains sequences homologous to the Escherichia coli  **rpoA** ,  **rpoB**  and rpoC genes.  Ref: 2381420 Mol Gen Genet, 1990
- Plastid transcription in the holoparasitic plant genus Cuscuta parallel loss of the rrn16 PEP  [ **RPOA** ]  promoter and of the  **rpoA**  and  **rpoB**  genes coding for the plastid encoded RNA polymerase.  Ref: 12624769 Planta, 2003
- RNA from this gene cluster is 30 fold more abundant than  **rpoB**  mRNA, suggesting that expression of  **rpoA**  is regulated at the level of translation or protein stability.  Ref: 1400453 J Biol Chem, 1992
- The 18 20 and 38 kDa polypeptides found in highly purified maize plastid RNA polymerase preparations are encoded by the maize plastid genes rpoC2,  **rpoB** , and  **rpoA** , respectively Hu, J. and Bogorad, L.  Ref: 2062657 Nucleic Acids Res, 1991
- and group 3, SigF to SigI and each RNAP core subunit  **RpoA** ,  **RpoB** , RpoC1 and RpoC2 were overproduced and purified from Escherichia coli cells, then polyclonal antibodies were prepared.  Ref: 12527296 J Mol Biol, 2003
- The RNA polymerase genes  **rpoA** ,  **rpoB** , and rpoD were mapped and appeared to be situated close together in one region.  Ref: 7590179 FEMS Microbiol Lett, 1995
- Chloroplast  **rpoA** ,  **rpoB** , and rpoC genes specify at least three components of a chloroplast DNA dependent RNA polymerase active in tRNA and mRNA transcription.  Ref: 3049574 J Biol Chem, 1988
- Promoters for NEP have been previously characterized in tobacco plants lacking PEP  [ **RPOA** ]  due to targeted deletion of  **rpoB**  encoding the beta subunit from the plastid genome.  Ref: 9701572 Plant Physiol, 1998
